# Impacts of hot and cold spells differ for acute and chronic ischaemic heart diseases

**DOI:** 10.1186/1471-2458-14-480

**Published:** 2014-05-21

**Authors:** Hana Davídkovová, Eva Plavcová, Jan Kynčl, Jan Kyselý

**Affiliations:** 1 Institute of Atmospheric Physics, Academy of Sciences of the Czech Republic, Prague, Czech Republic; 2 Faculty of Science, Charles University, Prague, Czech Republic; 3 Institute of Geophysics, Academy of Sciences of the Czech Republic, Prague, Czech Republic; 4 Centre for Epidemiology and Microbiology, National Institute of Public Health, Prague, Czech Republic; 5 Department of Epidemiology, Third Faculty of Medicine, Charles University, Prague, Czech Republic

**Keywords:** Epidemiology, Cardiovascular diseases, Mortality, Environment, Climate

## Abstract

**Background:**

Many studies have reported associations between temperature extremes and cardiovascular mortality but little has been understood about differences in the effects on acute and chronic diseases. The present study examines hot and cold spell effects on ischaemic heart disease (IHD) mortality in the Czech Republic during 1994–2009, with emphasis upon differences in the effects on acute myocardial infarction (AMI) and chronic IHD.

**Methods:**

We use analogous definitions for hot and cold spells based on quantiles of daily average temperature anomalies, thus allowing for comparison of results for summer hot spells and winter cold spells. Daily mortality data were standardised to account for the long-term trend and the seasonal and weekly cycles. Periods when the data were affected by epidemics of influenza and other acute respiratory infections were removed from the analysis.

**Results:**

Both hot and cold spells were associated with excess IHD mortality. For hot spells, chronic IHD was responsible for most IHD excess deaths in both male and female populations, and the impacts were much more pronounced in the 65+ years age group. The excess mortality from AMI was much lower compared to chronic IHD mortality during hot spells. For cold spells, by contrast, the relative excess IHD mortality was most pronounced in the younger age group (0–64 years), and we found different pattern for chronic IHD and AMI, with larger effects on AMI.

**Conclusions:**

The findings show that while excess deaths due to IHD during hot spells are mainly of persons with chronic diseases whose health had already been compromised, cardiovascular changes induced by cold stress may result in deaths from acute coronary events rather than chronic IHD, and this effect is important also in the younger population. This suggests that the most vulnerable population groups as well as the most affected cardiovascular diseases differ between hot and cold spells, which needs to be taken into account when designing and implementing preventive actions.

## Background

Heat waves and high temperature extremes have long been recognised to have serious impacts on human health. Mortality effects of heat waves and high ambient temperatures have been reported in many parts of the world, including North America [1,2], Europe [3,4], Asia [5,6] and Australia [7], and identification of population groups most at risk has become an important step towards better understanding effects of projected increasing occurrence of heat waves on human morbidity and mortality [8]. Recent studies in Europe have documented heat-related excess deaths both in large cities [9-11] and less urbanised regions (e.g. in Spain [12], Germany [13], and England and Wales [14]), mostly focusing on all-cause, cardiovascular and respiratory mortality. Increased vulnerability to heat has been found predominantly in the elderly [3,13], in females [12,15], and in persons with pre-existing diseases [10,16].

Studies regarding the impacts of low temperature extremes on human health have been less numerous [2,11], although effects of cold spells on mortality from cardiovascular disease (CVD) may be of at least similar importance as are those of hot spells [15]. In Europe, relationships have been found between cold exposure and organic-cause mortality [17], CVD mortality [11,15,18,19] and ischaemic heart disease (IHD) mortality [20-22]. While extreme cold episodes significantly increase mortality, impacts of low temperatures on health are more complex compared to those of heat waves, less direct, and confounded by such other factors as epidemics of influenza and acute respiratory infections [2,15,19,23].

Cardiovascular diseases, which comprise the largest proportion of total mortality and morbidity in developed countries [24], have widely been examined as to their association with excess mortality during high and low temperature extremes [2,11,16]. In most studies dealing with effects of thermal environment on cause-specific mortality, CVD was found to be particularly sensitive in both cold [18,25] and hot exposures [1,16]. Nevertheless, little attention has been devoted to date as to which particular CVDs are most affected by hot and cold spells. A few examples of such attempts are seen in recent studies by Gasparrini et al. [14], who specified pulmonary heart disease, heart failure, arrhythmias and atrial fibrillation as possible causes of increased cardiovascular risks in high temperatures, and Bhaskaran et al. [26], who reported that excess mortality in low temperatures could be due to acute myocardial infarction (AMI).

Ischaemic heart diseases comprise a major part of CVD mortality globally [24]. Despite a substantial decline over the last two decades, IHD remains the leading cause of death in the Czech Republic [27], and it accounted for 43% of all CVD deaths during 1994–2009. Elevated cold-related IHD mortality has been reported in the European population [20,21], and increased risk for heat-related IHD mortality has been documented in England and Wales [14], as well as in California [1]. However, none of the previous studies compared IHD mortality effects of hot and cold spells. Moreover, when analysing hot and cold spell effects on individual IHDs, one may compare the effects on acute fatal events (AMI mortality) and on deaths of those individuals with previous histories of IHD (chronic IHD mortality), which may differ in hot and cold exposures. This may point to physiological mechanisms manifested in heat- and cold-related health outcomes, and hence also to vulnerable population groups. Identification of those population groups would allow for development of better targeted and probably more efficient warning systems that can play an important role in reducing weather-related mortality [28,29].

The present analysis makes use of a recently completed national dataset and complements previous work concerning mortality associated with hot and cold spells in the population of the Czech Republic. Up to now, studies for the Czech population have dealt with all-cause mortality (e.g. Kyselý [30], Kyselý and Kříž [31]) or CVD mortality as a whole [15,19], because available data did not allow for more detailed analysis by examining individual diagnoses. Data covering the entire population of the Czech Republic since 1994 and including the detailed causes of death for all cases when IHD was cited as the primary cause have recently been released by the Czech Statistical Office and the Institute of Health Information and Statistics of the Czech Republic. This allows for studying individual IHDs and their association with hot and cold spells, a topic which, to our knowledge, has not been addressed in a comparative way (hot vs. cold effects, acute vs. chronic diseases) for any population. Such a study may yield new insight into heat and cold stress effects on cardiovascular health of vulnerable population groups.

## Methods

### Mortality data

Daily data on IHD mortality in the population of the Czech Republic (totalling 10.5 million as of 2009 and having changed only little since 1994) were collected and processed by the Czech Statistical Office and the Institute of Health Information and Statistics of the Czech Republic. The data cover the period 1994–2009. Each record includes the day of death, age at death, gender, region of residence, and primary cause of death according to the International Classification of Diseases (10th revision, ICD-10; used in the Czech Republic since 1994). The following ICD-10 codes were processed: all ischaemic heart diseases (I20–I25), acute myocardial infarction (I21–I22), and chronic ischaemic heart disease (I25).

Mortality due to IHD comprised 23% of all-cause mortality during 1994–2009 in the Czech Republic. A total of 400 063 deaths from IHD were recorded in the national registry during that period, with AMI (chronic IHD) accounting for 39.6% (59.1%) of those deaths. The remaining 1.3% consisted mainly of deaths from angina pectoris (I20) and other acute IHDs (I24) that are not analysed as separate groups owing to their small sample sizes (Table 1). The mortality database and trends in the rates of death from AMI and chronic IHD during 1994–2009 were described in detail in Davídkovová et al. [32].

**Table 1:** Numbers of ischaemic heart disease deaths in the Czech Republic over 1994–2009

	**Acute myocardial infarction**	**Chronic ischaemic heart disease**	**Angina pectoris**	**Other acute ischaemic heart diseases**	**All ischaemic heart diseases**
	**(I21–I22)**	**(I25)**	**(I20)**	**(I24)**	**(I20–25)**
**1994**	14834	15384	53	412	30683
**1995**	13772	16057	97	516	30442
**1996**	12797	14344	74	630	27845
**1997**	10108	15335	131	552	26126
**1998**	11697	12058	83	202	24040
**1999**	11847	12489	72	113	24521
**2000**	11347	11851	65	121	23384
**2001**	10665	12069	79	158	22971
**2002**	9807	12473	80	144	22504
**2003**	9237	12921	58	69	22285
**2004**	8083	12809	35	129	21056
**2005**	7352	15732	48	235	23367
**2006**	6871	15773	58	250	22952
**2007**	6667	19276	120	215	26278
**2008**	6789	18750	70	235	25844
**2009**	6677	18903	78	107	25765

Primary cause of death is according to the International Classification of Diseases (ICD-10).

### Standardisation of mortality data

**Figure 1:**
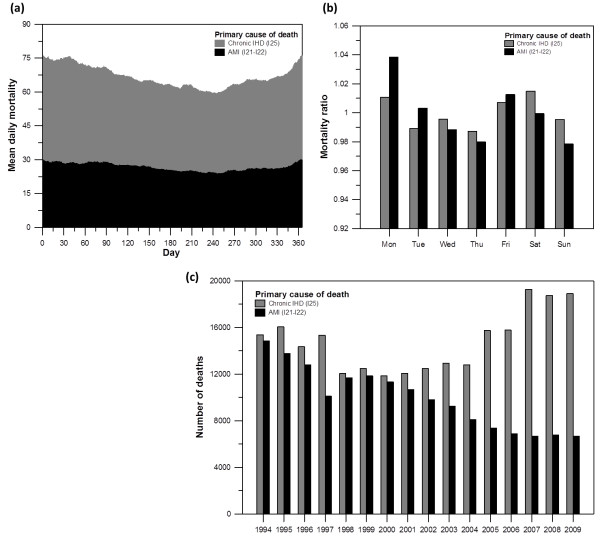
**Mean annual cycle (a), mean weekly cycle (b) and long-term changes (c) in mortality due to chronic ischaemic heart diseases (chronic IHD) and acute myocardial infarction (AMI) over 1994–2009, for the whole population.** The weekly cycle of mortality **(b)** is expressed by the ratio of the mean mortality on a given day to the overall mean mortality.

To remove the effects of long-term changes in mortality (related to demographic, health care, and lifestyle changes) as well as short-term variations due to annual and weekly cycles (Figure 1), the daily numbers of deaths must be standardised. Analogously to previous studies (e.g. [15]), series of daily excess mortality were established by calculating deviations of the observed and expected (baseline) mortality for each day of the examined period.

The expected number of deaths M_0_(y,d) for year y (y = 1994, … 2009) and day d (d = 1, … 365) was set according to

$$M_{0}\left(y,d\right)=M_{0}\left(d\right).W\left(y,d\right).Y\left(y\right)$$

where M_0_(d) denotes the mean daily mortality on day d in a year (computed from the mean annual cycle over 1994–2009, with epidemics of influenza/acute respiratory infections excluded from the data from which the mean annual cycle was determined, with a 7-day lag of mortality after epidemics; cf. [19,33]); W(y,d) is a correction factor for the observed weekly cycle of mortality, calculated separately for individual days of the week and defined as the ratio of the mean mortality on a given day to the overall mean mortality; and Y(y) is a correction factor for the observed year-to-year changes in mortality, defined as the ratio of the number of deaths in year y to the mean annual number of deaths during the analysed period. The correction factors for the weekly cycle W(y,d) and the year-to-year changes Y(y) were calculated over the April–November period when the effects of influenza/acute respiratory infections in the data are negligible.

A similar standardisation procedure (except for that epidemics had not been controlled for) had been used by, for example, Guest et al. [34], Whitman et al. [35], and Kyselý [30].

### Meteorological data

Daily air temperature data were provided by the Czech Hydrometeorological Institute. Mean temperature series were calculated by averaging data from 46 high-quality weather stations covering the area of the Czech Republic. The stations were selected so that they are representative for the area and population under study (the same methodology and the same set of stations was used in [15]). We employed mean daily air temperature as the input variable because it allows for using analogous definitions of hot and cold spells (see below), and because high-quality input variables needed for application of more complex biometeorological indices are available only for a small subset of the stations.

### Definitions of hot and cold spells

We use analogous definitions of hot and cold spells based on quantiles of the distribution of temperature anomalies as in our previous study for the same population and CVD mortality as a whole [15]. Hot and cold spells were defined as periods of at least two consecutive days with anomalies of average daily temperature from the mean annual cycle above the 90% quantile (below the 10% quantile); the quantiles were set from the empirical distribution of the anomalies over running 61-day periods centred on a given day of the year [15]. The threshold values of average daily temperature corresponding to the given quantiles of anomalies are approximately 23°C (−8°C) in mid-summer (mid-winter) while 20°C (−4°C) in the beginning and end of summer (winter). The 90%/10% quantile was set to delineate hot/cold days in preference to the 95%/5% quantile used in the previous studies [15,36], owing to the smaller sample sizes examined (the data were analysed with respect to individual diagnoses) and also due to the shorter time period of 1994–2009 for which the data were available. However, differences between results obtained with the 90%/10% quantile and the 95%/5% quantile are minor (not shown). Hot spells were analysed in summer (June–July–August, JJA) and cold spells in winter (December–January–February, DJF). A total of 35 hot spells and 37 cold spells were identified, and the average length of individual hot (cold) spell was 3.1 (3.8) days.

### Methods

Relative deviations of IHD mortality from the baseline were averaged over all hot/cold spells identified over 1994–2009, in sequences spanning 3 days before (D – 3) to 17 days after (D + 17) the onset of a hot/cold spell. This 3-week sequence comprises a relatively long period after the end of a hot/cold spell, in order to include possible lagged mortality effects. Statistical significance was evaluated by comparison with the 90% and 95% confidence interval (CI) around the zero line, estimated from the 2.5%, 5%, 95% and 97.5% quantiles of a distribution calculated by the Monte Carlo method. For each population group examined, the same numbers of 21-day sequences as the counts of the hot/cold spells were randomly drawn 10 000 times from the data over 1994–2009 in a given season, and corresponding quantiles were estimated. Periods in which mortality data were affected by epidemics of influenza/acute respiratory infections were excluded from all calculations.

## Results

### Effects of hot and cold spells on IHD mortality

**Figure 2:**
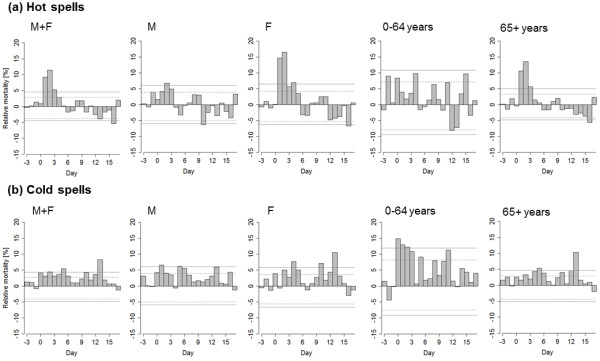
**Mean relative deviations of ischaemic heart disease mortality for 21-day sequences spanning 3 days before to 17 days after the onset of hot spells (a) and cold spells (b) for the whole population (M + F), males (M), females (F), younger population (0–64 years) and the elderly (65+ years).** Solid (dashed) lines denote the 2.5% and 97.5% (5% and 95%) quantiles of deviations obtained by the Monte Carlo method.

Relationships between hot and cold spells and IHD mortality in the whole population, males, females, younger age group (0–64 years) and the elderly (65+ years) are shown in Figure 2. Both hot and cold spells were associated with excess IHD mortality, with different magnitude, duration and lag of the effects. For hot spells and the population as a whole, IHD mortality increased markedly from day D + 1 to D + 4, with peak on D + 2. For cold spells, by contrast, the excess IHD mortality was less significant on individual days but persisted for a longer period (up to almost two weeks after cold spell onset; significant on most days D + 0 to D + 13 in the whole population). We note that excess mortality on days around D + 10 for cold spells is due to lagged effects, not direct exposure to cold, as mean temperature anomalies become close to zero around 9 days from the beginning of cold spells [15].

Hot and cold spells were linked to excess IHD mortality in both male and female populations. During hot spells, much larger increase in IHD mortality was found for females (more than 15% excess mortality on D + 2) compared to males, and in the elderly. The effect of cold spells on IHD mortality was comparable in women and men as to the magnitude of excess mortality, with a tendency towards longer lags in women.

The effects of cold spells on IHD mortality were more direct and more pronounced in the younger age group (0–64 years); on four consecutive days after the onset of a cold spell (from D + 0 to D + 3), mean relative excess mortality exceeded 10%. By contrast, effects of extreme heat on IHD mortality in this age group were much less pronounced.

We did not find any dependence of the excess IHD mortality on intensity (measured by mean temperature) or duration of a hot/cold spell.

### Comparison of impacts of hot and cold spells on AMI and chronic IHD mortality

**Figure 3:**
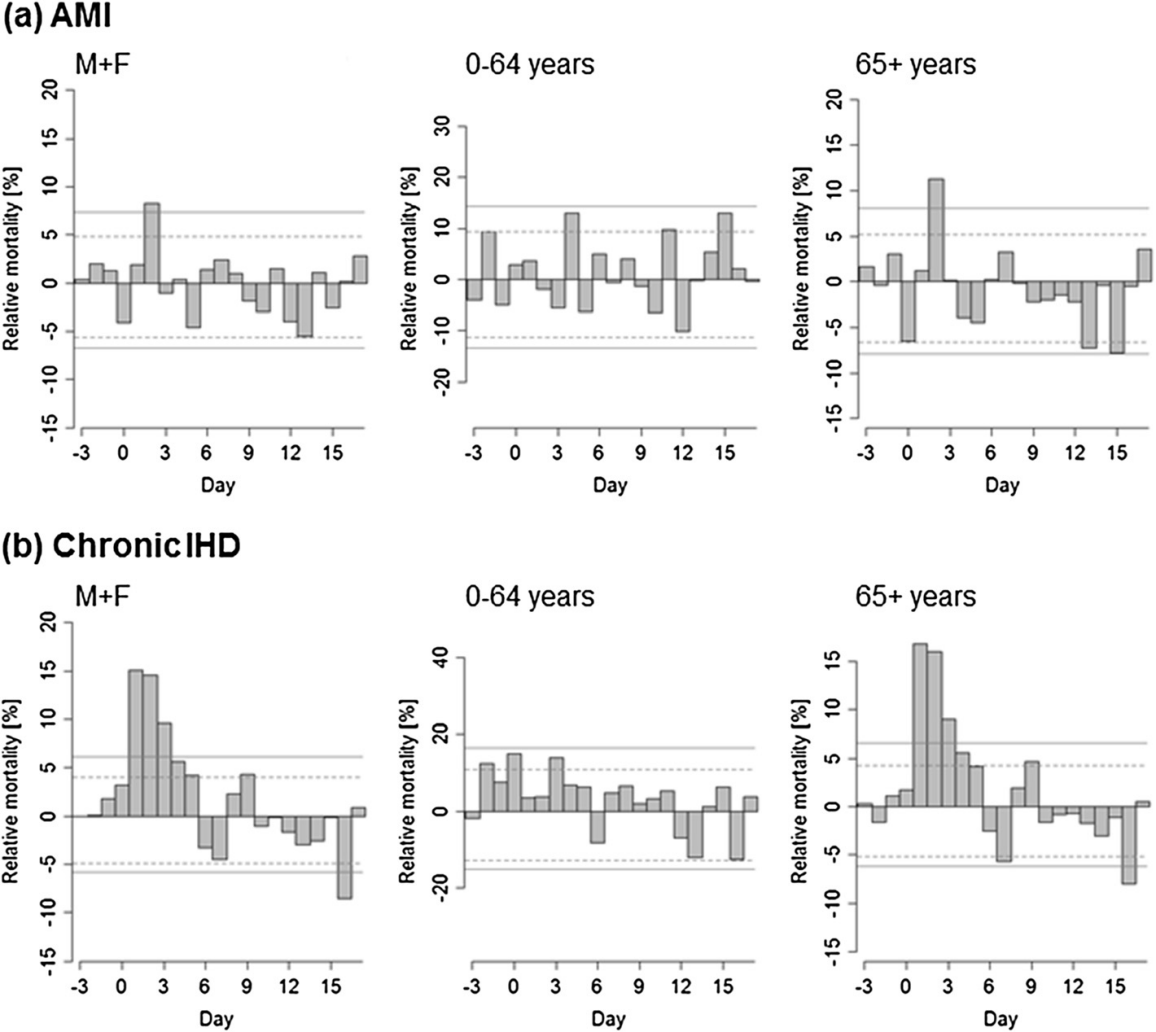
**Mean relative deviations of mortality for acute myocardial infarction (AMI, a) and chronic ischaemic heart disease (chronic IHD, b) in hot spells.** The range of y-axis is adjusted so that the width of confidence bounds is visually the same in all plots. Other details as in Figure 2.

**Figure 4:**
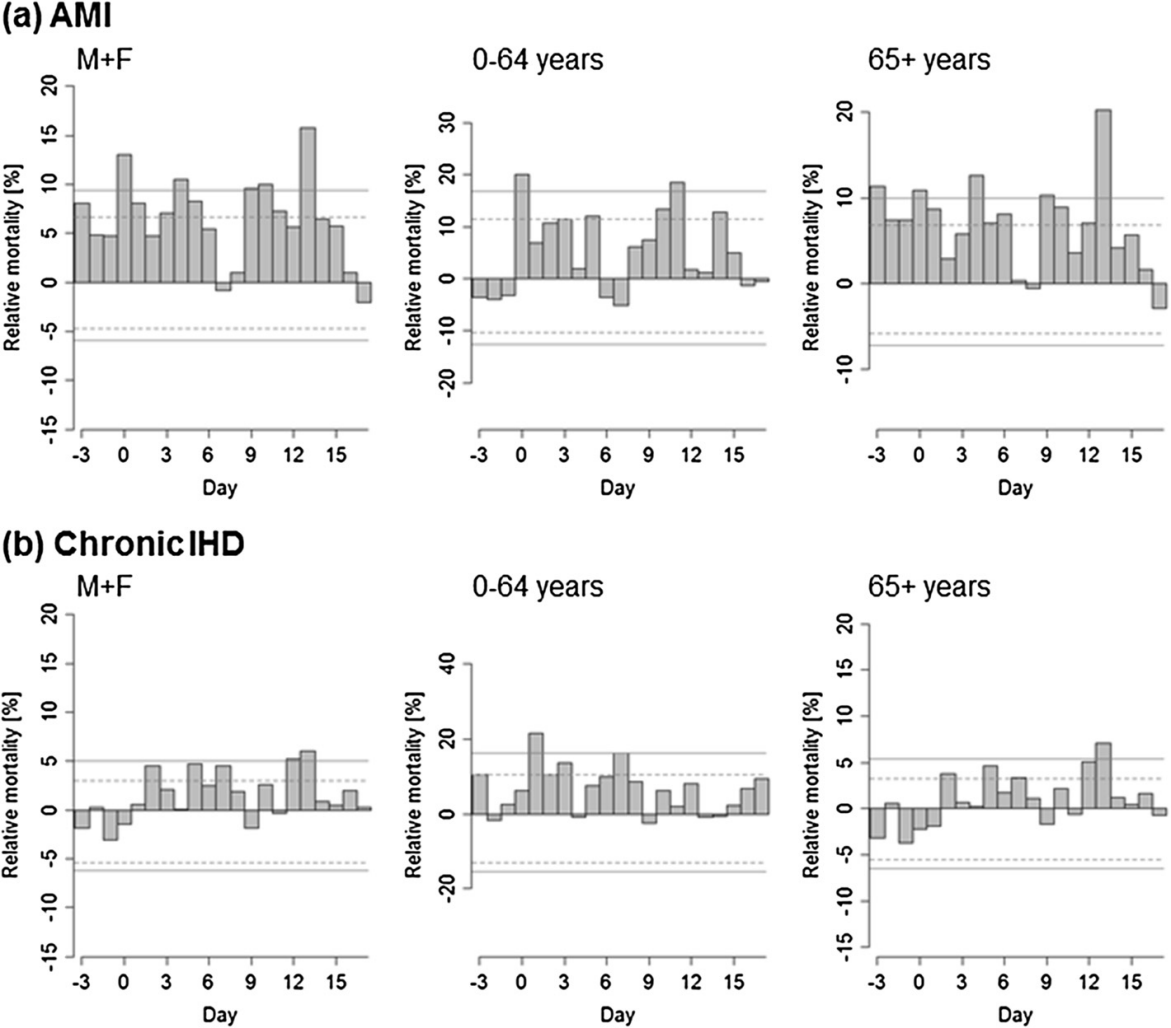
**Mean relative deviations of mortality for acute myocardial infarction (AMI, a) and chronic ischaemic heart disease (chronic IHD, b) in cold spells.** Other details as in Figures 2 and 3.

Effects of hot and cold spells on mortality from AMI and chronic IHD in the population as a whole, the younger age group (0–64 years), and the elderly (65+ years) are shown in Figures 3 and 4. For hot spells, the patterns for acute and chronic IHD are clearly different (Figure 3). Mortality due to chronic IHD increased sharply on the first day after the onset of a hot spell (excess mortality about 15% on D + 1) and high excess mortality persisted for 5 days, whereas excess mortality from AMI was significant on a single day only (D + 2) and the increase was much lower (excess mortality about 8%) compared to chronic IHD mortality.

In contrast to hot spells, the mortality impacts of cold spells were more pronounced for AMI than chronic IHD (Figure 4). The differences between AMI and chronic IHD were manifested mainly in the elderly while they were relatively minor in the younger population. Mortality due to AMI was elevated in both age groups and the effect of cold was immediate (excess mortality of 20% on D + 0 in the younger age group), whereas excess chronic IHD mortality was observed predominantly in the younger age group and was more lagged (mortality increased by more than 20% on day D + 1). Excess AMI mortality occurring already 3 days before the beginning of a cold spell is probably related to typical weather patterns on days preceding the onset of a cold spell (when temperatures may already be below normal).

**Figure 5:**
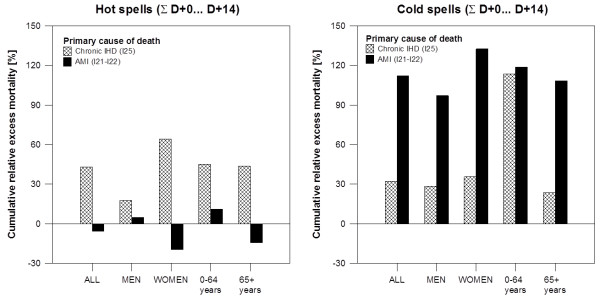
Cumulative relative excess mortality (∑D + 0… D + 14) for acute myocardial infarction (AMI) and chronic ischaemic heart disease (chronic IHD) in hot spells (left) and cold spells (right) in different population groups.

To compare the average effects of hot and cold spells on acute and chronic IHD mortality, we computed cumulative excess mortality by summing mean relative excess deaths from D + 0 to D + 14 for hot and cold spells (Figure 5). For hot spells, much larger cumulative excess mortality was observed for chronic IHD compared to AMI in all examined population groups. On the contrary, for cold spells, cumulative excess AMI mortality substantially exceeded IHD mortality in all population groups, except for the younger age group (0–64 years) where the difference was small. Plausible modifications of the periods over which mean cumulative excess mortality is summed for hot and cold spells do not affect this contrasting pattern. These results also suggest that the IHD mortality effects of a cold spell are on average considerably larger than those associated with a hot spell. In the population as a whole, the estimated excess mortality associated with an average hot spell is ~40% of daily mortality while the excess mortality associated with an average cold spell is ~140% of daily mortality (Figure 5). We note that for hot spells, the cumulative excess mortality over days D + 0 to D + 14 reflects also the mortality displacement effect; however, if mean excess mortality is summed over days D + 0 to D + 4 only, when mortality deviations are positive (Figure 2), the estimate of excess mortality associated with an average hot spell rises only slightly (~50% of daily mortality). Given that the number of hot and cold spells is comparable (see above) and the baseline daily IHD mortality is higher in winter than summer (Figure 1), the estimates suggest that cold spells were associated with 3 to 4 times more excess deaths due to IHD compared to hot spells.

## Discussion

### Hot and cold spell effects on IHD mortality

The results show that both hot and cold spells have significant impacts on IHD mortality, but differences were found between genders and age groups. In hot spells, the peak excess IHD mortality was much larger while the duration of the effects of heat on IHD mortality was shorter and concentrated on days with elevated ambient temperatures. Impacts of cold spells on IHD mortality were less pronounced and persisted for a longer period after the end of a cold spell.

With respect to gender, heat-related excess IHD mortality was much larger in women than in men, while excess IHD mortality associated with cold spells was less significant and more lagged in females compared to males. A number of studies have shown that women are more vulnerable to heat than are men [10,12,15], while gender-related differences in cold-related mortality are less understood [37,38]. Greater vulnerability of females to heat is probably related to older mean age and pre-existing chronic diseases, as discussed in detail, for example, by Hajat et al. [39], Kyselý and Kříž [31], and Schneider et al. [28].

For winter cold spells, larger relative excess IHD mortality was observed in the younger age group (0–64 years). In the elderly, effects of cold exposure were more lagged, with the IHD mortality observed to peak several days after the end of a typical cold spell. This finding is consistent with results from the previous study for aggregated CVD mortality showing that low temperature extremes affect cardiovascular health more markedly in the middle-aged population compared to the older age groups [19].

Several physiological mechanisms could play a role in IHD’s meteorological sensitivity. Exposure to extreme temperatures could induce changes in contractility of veins, blood viscosity and blood pressure, and it may lead to altered blood coagulation [37]. Consequent changes in heart function may result in myocardial ischemia and could cause acute cardiovascular events such as AMI, particularly in people with pre-existing chronic diseases [28].

### Different impacts of hot and cold spells on AMI and chronic IHD mortality and possible physiological mechanisms

Both high and low temperature extremes were linked to excess mortality for AMI and chronic IHD but different patterns were found, thus suggesting different physiological mechanisms playing dominant roles in extreme heat/cold exposures.

#### AMI mortality in hot and cold spells

Significant excess AMI mortality was associated predominantly with low temperatures and persisted up to almost two weeks after the beginning of a cold spell, while the effects of hot spells on AMI mortality were much weaker and significant only on a single day (D + 2).

A similar pattern was recently reported in England and Wales by Bhaskaran et al. [26]. They found increasing incidence of non-fatal AMI associated with cold exposure and no risk of AMI associated with heat. Moreover, effects of cold exposure were observed from 2 to 14 days after decrease of temperature [26], which is consistent with our results for Central-European population. A study from Germany also documented lagged effects of low temperatures on non-fatal AMI and more direct effect of cold on fatal AMI [38]. An association between low temperature and higher incidence of AMI was recently reported also in the Netherlands [40]. These findings suggest that changes in thermoregulation induced by cold ambient temperatures may cause severe deterioration in health, leading to acute coronary events and death in a short time. The elderly population and people with histories of previous IHD have been shown to be most at risk of AMI in the cold [26]. Moreover, cold-related cardiovascular symptoms such as arrhythmias and chest pain have been found predominantly in elderly people with pre-existing coronary heart disease or cardiac insufficiency [41]. In our study, the effects of cold exposure on AMI mortality were observed in both age groups, and larger excess AMI mortality at the beginning of a cold spell was seen in the younger population than in the elderly. Younger age and higher cholesterol levels have been reported as risk factors for AMI during unusually cold winter in a study from Northern Europe [42], documenting an increase in incidence of acute coronary angiographies with a mean temperature decrease of 7.5°C between a warm winter and a cold winter. These findings suggest that cold exposure is a triggering factor for acute cardiac events, with younger people being more vulnerable.

#### Chronic IHD mortality in hot and cold spells

The results further suggest that the presence of chronic IHD increases mortality risk associated with extreme heat more than for extreme cold. During hot spells excess mortality due to chronic IHD was much larger than excess AMI mortality. Women and the elderly population (65+ years) were most at risk of dying from chronic IHD during heat exposure. The findings confirm the previously reported results that excess deaths during hot spells are mainly among people with chronic diseases whose health has been compromised before the hot spell [10,16]. The impact of hot weather on cardiovascular health is unlagged and may cause severe deterioration of health leading to death in a short time, especially in those people with chronic CVD. In extreme heat, an increase in blood viscosity and cardiac output followed by hypotension, dehydration and renal failure could result in thromboembolic disease, malignant cardiac arrhythmias and sepsis-like shock leading to death [43,44].

In cold spells, excess mortality due to chronic IHD was more lagged and less significant. A considerably elevated mortality due to chronic IHD was observed in the younger age group (0–64 years), while in the elderly effects of cold exposure on chronic IHD mortality were insignificant. Exposure to cold may lead to death from acute events rather than from chronic IHD in the elderly. Cold stress could trigger symptoms of angina pectoris, cardiac arrhythmias and chest pain in stable IHD [41], caused by such cold-related changes as vasoconstriction and/or increases in blood pressure, blood viscosity, red blood cell counts and plasma cholesterol [45]. Together with heightened haemoconcentration and elevated coagulation potential, these changes could lead to thrombosis and plaque rupture resulting in acute coronary event [46]. Therefore, the risk of acute ischaemic event is increased in the cold, particularly in people with pre-existing IHD.

### Mortality displacement effect

The results also show declines in mortality after hot spells, while similar effect was not observed for cold spells (Figure 2). The fact that the overall mortality impacts of hot spells are reduced due to a displacement effect has been documented in many studies (e.g. [30,47-49]); the peak of heat-related deaths is followed by a period of up to 3 weeks with negative deviations of mortality. In the present study, the harvesting effect of individual hot spells was not considered and all spells identified according to the given definition were involved in the analysis. The short-term mortality displacement, observed after the peak in heat-related deaths (Figure 2), points to the presence of very susceptible persons for which the heat exposure precipitates death. The results further showed that despite the presence of harvesting, excess mortality for chronic IHD was found in all examined population groups when considering the period of two weeks after a hot spell as a whole (Figure 5).

### Limitations of the study

We note that data based on death certificates may contain non-negligible levels of noise, and this may be the case especially for IHD for which it is often difficult to discern between AMI and chronic IHD as the primary cause of death. It is plausible that many deaths from AMI, especially among older persons who die out of hospital, are coded as chronic IHD while deaths from AMI occur in persons who already had pre-existing IHD. This is a limitation for any study of IHD mortality based on death certificates. However, the autopsy rate in the Czech Republic is among the highest in Europe and changed little over the study period (from 34% in 1994 to 29% in 2009) [32], which suggests that the quality of the national mortality register data is at least comparable to western-European countries. While negative or insignificant results (as to differences between effects of temperature extremes on acute and chronic IHD) would not necessarily mean that the effects do not exist (they may be masked by the noise), the observed clear pattern of differences between hot and cold spell effects on acute and chronic IHD – found in spite of the data limitations – cannot be interpreted as an artefact due to possible errors in death certificates. Different impacts of temperature extremes on acute and chronic CVD have been found also for the UK population [14,26], and studies for other regions with available data are needed.

Effects of the air pollution on mortality were not controlled for since the analysis was carried out for a population majority of which lives in cities with less than 50 000 inhabitants or in a rural environment, while only a small portion (21%) in large cities with more than 100 000 inhabitants [50]. This also makes the analysis different from the majority of other studies on heat-related mortality, which usually deal with urban populations. We do not suggest that the effects of air pollution are negligible in a population with a relatively small level of urbanization; however, it is difficult to collect high-quality and homogeneous air pollution data that may be representative for such population living in very diverse environments. While ambient temperature anomalies are usually similar for distances of many dozens or even hundreds km, air pollution data – due to local effects such as heating, transportation, local industrial and agricultural activities, etc. – may not be representative for distances exceeding few hundred meters. Focusing on urban population only (the city of Prague) would decrease the size of the mortality data samples by an order of magnitude, and hence lessen the significance of results. Anyway, the issue of regional differences in the effects of hot and cold spells, which may be related to environmental as well as socio-economic factors, is an important topic attracting growing interest worldwide and also subject of ongoing research in Central Europe [13,51].

## Conclusions

The results yield new insight into links between temperature extremes and mortality due to acute and chronic forms of IHD. We show that both hot and cold spells were associated with excess IHD mortality in the Czech Republic, but the most affected population groups differed and the excess mortality was due to different prevailing health outcomes for heat and cold. In hot spells, increases in IHD mortality were most pronounced in the elderly (65+ years) and in females, while in cold spells, significant excess IHD mortality was found also in the younger age group (0–64 years). For summer hot spells, the largest excess mortality was related to chronic IHD while the increase in mortality from AMI was much smaller. For winter cold spells, by contrast, impacts were observed mainly for AMI mortality.

Different patterns in the mortality effects of hot and cold spells observed for AMI and chronic IHD suggest several different mechanisms involved in physiological processes leading to excess deaths. Prolonged exposure to heat stress may result in thermoregulatory failure followed by heat-related disorders (such as hyperthermia, dehydration, hypotension, heat exhaustion and consequent renal failure) leading to cardiovascular complications resulting in death, and mainly in those individuals with pre-existing IHD. On the other hand, cold-related deaths are associated predominantly with acute cardiac events, irrespective of age group and gender, most probably due to changes in blood coagulation that result in thrombosis during cold stress. Better understanding of those risk factors and physiological mechanisms playing roles in the development of cardiovascular problems in extreme temperatures could help identify individuals most at risk and better focus preventive actions, including biometeorological forecast and alerts*.*

The results of studies on temperature–mortality relationships are difficult to compare due to differences in study designs, characteristics of datasets and methodology, including definitions of hot and cold spells and how possible confounding effects (such as epidemics of influenza/acute respiratory infections) are addressed. This underlies the need for further comparative studies dealing with the effects of both hot and cold spells on cause-specific mortality in different countries and climates that are directed to improving prevention strategies for reducing the mortality risk in extreme temperatures. Nevertheless, in spite of differences in study designs and methods, the emerging pattern of different impacts of temperature extremes on acute and chronic cardiovascular diseases has been found for populations living in different climatic and socio-economic conditions (the UK, Central Europe).

Rising mean summer temperatures are very likely to lead to an increase in the frequency, duration and severity of heat waves in future [52,53], and, even in a warming climate, intensity and duration of extreme cold events may persist into the late 21st century [54]. This suggests that both heat waves and cold spells will represent major public health concerns, with impacts probably exacerbated due to the population’s ageing and increasing level of urbanisation. Better understanding of the observed heat- and cold-related effects on cardiovascular health is an essential step towards understanding how climate change may modify these effects, and, as an ultimate goal, towards designing and implementing efficient measures to reduce the negative consequences on public health of both types of extremes.

## Abbreviations

IHD: Ischaemic heart disease; AMI: Acute myocardial infarction; CVD: Cardiovascular disease.

## Competing interests

The authors declare that they have no competing interests.

## Authors’ contributions

HD and JKys participated in the design of the study and wrote the manuscript. HD researched the data and drafted the manuscript. EP was responsible for most statistical analyses. All authors participated in interpretation of the data and reviewed/edited the manuscript. All authors read and approved the final version of the manuscript.

## Pre-publication history

The pre-publication history for this paper can be accessed here:

http://www.biomedcentral.com/1471-2458/14/480/prepub
